# Predicting Axial Impairment in Parkinson’s Disease through a Single Inertial Sensor

**DOI:** 10.3390/s22020412

**Published:** 2022-01-06

**Authors:** Luigi Borzì, Ivan Mazzetta, Alessandro Zampogna, Antonio Suppa, Fernanda Irrera, Gabriella Olmo

**Affiliations:** 1 Department of Control and Computer Engineering, Politecnico di Torino, 10129 Turin, Italy; gabriella.olmo@polito.it; 2 Polito^BIO^Med Lab—Biomedical Engineering Laboratory, Politecnico di Torino, 10129 Turin, Italy; 3 Department of Information Engineering, Electronics and Telecommunication, Sapienza University of Rome, 00184 Rome, Italy; ivan.mazzetta@uniroma1.it (I.M.); fernanda.irrera@uniroma1.it (F.I.); 4 Department of Human Neurosciences, Sapienza University of Rome, 00185 Rome, Italy; alessandro.zampogna@uniroma1.it (A.Z.); antonio.suppa@uniroma1.it (A.S.); 5 IRCCS NEUROMED Institute, 86077 Pozzilli, Italy

**Keywords:** wearable sensors, machine learning, feature extraction, dimensionality reduction, freezing of gait (FOG), gait, postural instability and gait difficulty score (PIGD), time up and go, Levodopa, Parkinson’s disease

## Abstract

Background: Current telemedicine approaches lack standardised procedures for the remote assessment of axial impairment in Parkinson’s disease (PD). Unobtrusive wearable sensors may be a feasible tool to provide clinicians with practical medical indices reflecting axial dysfunction in PD. This study aims to predict the postural instability/gait difficulty (PIGD) score in PD patients by monitoring gait through a single inertial measurement unit (IMU) and machine-learning algorithms. Methods: Thirty-one PD patients underwent a 7-m timed-up-and-go test while monitored through an IMU placed on the thigh, both under (ON) and not under (OFF) dopaminergic therapy. After pre-processing procedures and feature selection, a support vector regression model was implemented to predict PIGD scores and to investigate the impact of L-Dopa and freezing of gait (FOG) on regression models. Results: Specific time- and frequency-domain features correlated with PIGD scores. After optimizing the dimensionality reduction methods and the model parameters, regression algorithms demonstrated different performance in the PIGD prediction in patients OFF and ON therapy (r = 0.79 and 0.75 and RMSE = 0.19 and 0.20, respectively). Similarly, regression models showed different performances in the PIGD prediction, in patients with FOG, ON and OFF therapy (r = 0.71 and RMSE = 0.27; r = 0.83 and RMSE = 0.22, respectively) and in those without FOG, ON and OFF therapy (r = 0.85 and RMSE = 0.19; r = 0.79 and RMSE = 0.21, respectively). Conclusions: Optimized support vector regression models have high feasibility in predicting PIGD scores in PD. L-Dopa and FOG affect regression model performances. Overall, a single inertial sensor may help to remotely assess axial motor impairment in PD patients.

## 1. Introduction

Parkinson’s disease (PD) is a neurodegenerative disorder clinically characterized by bradykinesia, tremor, and rigidity [1]. Besides these cardinal signs, axial impairment, including gait and postural disorders, is among the most disabling symptoms responsible for progressive motor impairment and frequent falls in PD [2,3]. According to the Hoehn and Yahr scale, the staging of PD is based on the severity of axial signs, including balance impairment and the ability to walk independently [4]. Currently, the clinical assessment of axial impairment in PD implies the measurement of the postural stability/gait difficulty (PIGD) score which represents an accurate indicator of the disease severity and prognosis [5]. To obtain quantitative markers of disease progression, it would be relevant to measure the PIGD score objectively in patients with PD (PDPs).

Over the last two decades, wearable sensing systems based on accelerometers and gyroscopes have been increasingly used for the objective monitoring of gait and balance in PDPs [6,7,8]. These technologies have provided highly accurate data on gait and balance in PD [9,10], through the analysis of inertial data recorded during different activities, including stance [11,12], postural transitions [13,14], walking [15,16,17], and turning [18,19]. In addition, several machine-learning (ML) algorithms have been previously used to objectively assess PDPs and evaluate their disease severity [20,21]. However, so far, only a few studies have used sensor-based recordings in PD to predict specific Unified Parkinson’s Disease rating scale (UPDRS) items concerning balance and gait or even the PIGD score, reaching suboptimal performances. In [22], inertial data from 75 PDPs were recorded during gait, using a single IMU on waist. A support vector machine (SVM) classifier was employed for gait detection, and the power spectra in the 0–10 Hz range was computed and used as output. Results showed that the algorithm output was correlated with the UPDRS gait (*r* = −0.73, *p* < 0.001) and postural stability (*r* = −0.42, *p* < 0.001) scores. In [23], 31 PDPs were equipped with 3 inertial sensors on the lower back and on each foot. Inertial data were recorded in home environments, and gait, turn, and stance activities were detected and analysed. Several measures regarding quantity and quality of movements were extracted, selected, and input to a multivariable linear regression model. The algorithm outcome correlated with the total UPDRS-part III (*r* = 0.48, *p* = 0.007) and with the PIGD score (*r* = 0.61, *p* < 0.001). These studies have not considered several clinical biases with relevant impact on sensor-based measures such as the effect of L-Dopa and freezing of gait (FOG) occurrence. In addition, the use of multiple inertial sensors reduced the unobtrusiveness and comfortableness of adopted sensing systems [23].

In this study, axial impairment in PD was assessed by calculating the PIGD scores through sensor-based technologies. First, the impact of L-Dopa on ML models’ performance for the PIGD prediction was examined by comparing patients under (ON) and not under (OFF) dopaminergic therapy. In addition, patients with and without FOG were compared to consider possible differences in the PIGD prediction based on the occurrence of FOG. To limit the sensing system obtrusiveness, a single small and lightweight inertial sensor was used. To improve the ecological value of our data, the wearable sensor was attached to the thigh of the patients thus resembling a common smartphone placed in the front pocket. Still, to optimize the sensing performances and improve the accuracy, many time- and frequency-domain features were computed, along with the classical spatiotemporal gait parameters, and ML algorithms were used, owing to their ability to achieve automatic storage, elaboration, and interpretation of a large amount of data driven by wearable sensors [24,25]. The objective and automatic evaluation of the PIGD score based on a single wearable sensor would open new opportunities also in the remote assessment of the disease progression in PD in line with new telemedicine approaches. Indeed, although the International Parkinson and Movement Disorder Society has recently validated standardised procedures and scales to remotely assess PDPs, these telemedicine procedures do not allow the evaluation of axial impairment which requires specific clinical tests. Hence, predicting the PIGD score through a single inertial sensor and dedicated ML algorithms would allow obtaining more ecological data reflecting disease progression in PDPs.

The rest of this paper is organized as follows: Section 2 describes the population enrolled in this study, the experimental protocol, and the processing performed on inertial data; Section 3 reports the results obtained, further evaluating the effect of L-Dopa and FOG; in Section 4, the results are discussed, while in Section 5, conclusions are drawn and future works are proposed.

## 2. Materials and Methods

### 2.1. Subjects

Thirty-one PDPs were enrolled from the Movement Disorder outpatient clinic of the Department of Human Neurosciences, Sapienza University of Rome, Italy, based on the following inclusion criteria: diagnosis of idiopathic PD; lack of dementia (i.e., Mini-Mental State Examination—MMSE > 24); ability to walk autonomously; lack of neurological, orthopaedic or rheumatic comorbidities possibly affecting gait. Two neurologists, with expertise in movement disorders, investigated the presence of FOG in all patients by a clinical interview and direct physical examination. Overall, 17 PDPs showed definite FOG (*FOG+*), while the remaining 14 never experienced FOG (*FOG−*). The following standardised scales for clinical assessment were used: Hoehn and Yahr scale (H&Y), modified Movement Disorder Society—unified Parkinson’s disease rating scale (MDS-UPDRS) part III, FOG questionnaire (FOG-Q), MMSE, frontal assessment battery (FAB), Hamilton depression rating scale (HAM-D), and Beck anxiety inventory (BAI). In order to further assess axial impairment, the PIGD score was calculated, measured as the sum of items 2.12, 2.13, 3.10, 3.11, and 3.12 of MDS-UPDRS, both OFF and ON state of therapy. Patients were clinically assessed both OFF (i.e., after L-Dopa withdrawal for at least 12 h) and ON (1 h after L-Dopa intake) state of therapy. In addition, L-Dopa equivalent daily doses (LEDDs) were calculated for each patient, according to standardised procedures [26]. Demographic and clinical features of PDPs enrolled in this study are summarized in Table 1 and Table 2. Experimental procedures were approved by the institutional review board and performed according to the Declaration of Helsinki.

### 2.2. Experimental Protocol and Data Acquisition

**Figure 1:**
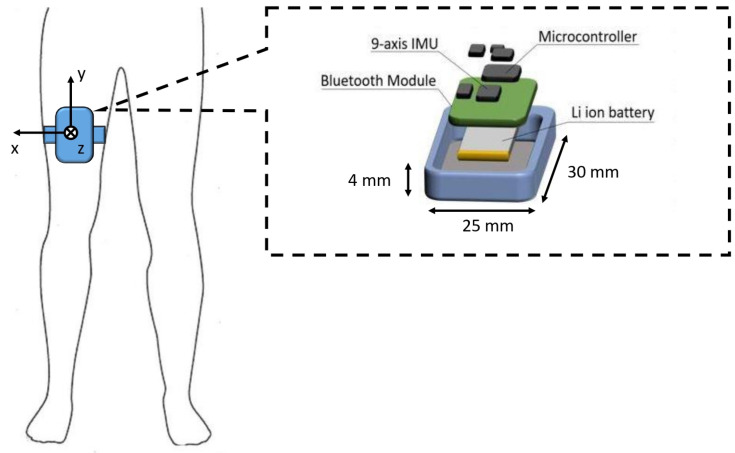
Sketch of sensor position, together with an exploded view of the neMEMSI device.

Patients were asked to perform a 7-m timed-up-and-go (TUG) test consisting of the following procedures: (1) getting up from a chair; (2) walking in a straight line for 7 m; (3) turning; (4) walking back; (5) sitting down. To maximize the ecological value of our recordings and trigger the possible occurrence of FOG, the 7-m TUG test was performed in a free living-like environment with a number of factors simulating a domestic setting (e.g., passage from a spacious room to a narrow and furnished corridor with the interposition of an open door) [8]. Patients’ gait was video-recorded through a camera and monitored by a single IMU placed and fixed on the thigh through an elastic band (Figure 1). The IMU positioning on the patient’s thigh was implemented so that when the patient was standing, the *y*-axis represented the inverse gravity vector and *x*-axis lies in the frontal plane. Hence, the angular velocity around the *x*-axis allowed a good representation of the thigh motion during linear gait. The STMicroelectronics system-on-board prototype neMEMSi [27] was equipped with the following components: a 9-axis IMU (LSM9DS0), integrating a 3-axis accelerometer and a 3-axis gyroscope; a Bluetooth V3.0 module (BT33); a lithium-ion battery; an ultralow-power 32-bit microcontroller (STM32L1). Sensors range was settable up to ±16 g and ±2000 dps for accelerometer and gyroscope, respectively. A sample frequency up to 200 Hz can be used. The device size (including battery) is 25 mm × 30 mm × 4 mm (Figure 1). Moreover, neMEMSi includes a temperature sensor, a hygrometer sensor and a pressure sensor that were not used for this study. Table 3 reports technical characteristics of the inertial sensors embedded in the IMU (specifications refer to those set in this study). Before placement, a preliminary conventional calibration of the inertial sensors was performed, including software correction of the displacement of the IMU framework with respect to the earth framework. Specifically, static acquisitions of both accelerometer and gyroscope data were carried out as indicated in [28,29]. The IMU was systematically arranged in specific positions on a table. The operations to correct or align the sensor with the reference framework were performed in real-time, with NeMEMSi transmitting data via Bluetooth to the PC. Orientation was derived from measurements and compared to earth observation framework. Rotation between sensor and earth quaternions was calculated at each IMU tested position and used for orientation correction. Once the calibration procedure was finished, the IMU was positioned on the patient. The resulting data were sent in real-time to a personal computer through the neMEMSi Bluetooth module and progressively saved in CSV format. Each CSV file was related to a single test. Data in CSV files were processed offline as described in the next section.

### 2.3. Preprocessing

In this section, the signal processing steps performed prior to the statistical analysis and the regression task are described. First, a sensor fusion process was performed to compute the orientation signal from the raw accelerometer and raw gyroscope readings (Section 2.3.1). Then, the orientation signal was used to detect walking bouts from the entire TUG recording (Section 2.3.2). Finally, inertial data were segmented and temporal and spectral features were extracted from each stride (Section 2.3.3).

#### 2.3.1. Orientation Estimation

**Figure 2:**
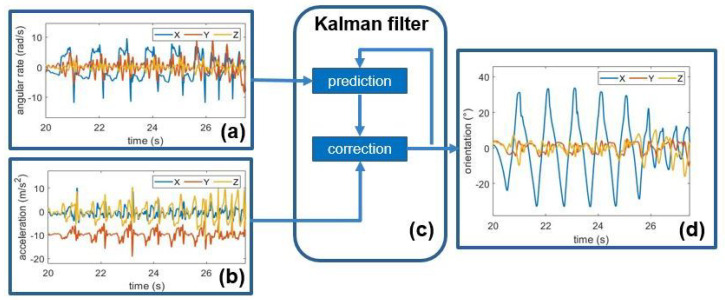
Schematic of the Kalman filter, together with input and output. Raw gyroscope (**a**) and accelerometer (**b**) readings are input to the Kalman filter (**c**) to provide an estimate of orientation (**d**).

A Kalman filter [30] was used to estimate the sensor orientation from the fusion of raw acceleration and angular velocity recordings. The sensor fusion algorithm alternates iteratively two processes, including a prediction step and a correction step. The former consists in an approximation of the orientation estimate, performed through an integration of the gyroscope readings; the latter exploits accelerometer readings to correct the drift due to the integration of the slow-varying bias affecting the gyroscope measurements [31]. Figure 2 shows the raw gyroscope (a) and raw accelerometer (b) readings, and the orientation estimate (d) obtained using the Kalman filter (c). After orientation estimation, acceleration, angular velocity, and orientation signals were filtered, in order to keep only the frequency components of interest while removing mean values, low-frequency trends, and high frequency noise. A second-order zero-lag band-pass Butterworth filter was used to keep only components in the 0.5–20 Hz band, while avoiding phase distortion.

#### 2.3.2. Walking Bouts Detection

In order to select only the walking segments of data, a continuous wavelet transform (CWT)-based approach was implemented, which is often used for walking steps detection algorithms [32,33]. CWT uses inner products to measure the similarity between the signal x(t) and an analysing function, which is a wavelet ψ. Equation (1) reports the formula for CWT computation. First, the wavelet is shifted by b∈R values and stretched/compressed by $a\in R^{+}$ values, then the shifted and stretched/compressed versions of the wavelet $\psi^{*}\left(\frac{t-b}{a}\right)$ is compared to the signal x(t) in order to compute their similarity. This procedure is performed using a mother wavelet ψ and all possible values of *a* and *b*.

(1) $$X\left(a,b,x\left(t\right),\psi \right)\;=\;\int_{-\infty }^{+\infty }x\left(t\right)\frac{1}{a}\psi^{*}\left(\frac{t-b}{a}\right)dt$$

In this study, a *Morse* mother wavelet was used, due to its similarity with the orientation signal pattern during walking. Moreover, the scale parameter (*a*) was set so that the frequency analysis was performed in the range 0.5–2 Hz. This is done considering that stride time is rather heterogeneous in PDPs, due to the variability of motor features among patients [18], the pharmacological condition [8], and the gait velocity [34]. In [35], stride time in PD was found to be 1.13 ± 0.21 s, taking into consideration eleven studies on parkinsonian gait.

**Figure 3:**
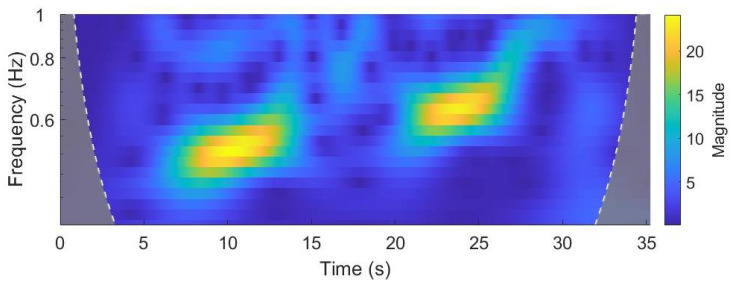
Absolute value of the CWT plotted as a function of time and frequency. Yellow zones represent the walking segments of the signal.

**Figure 4:**
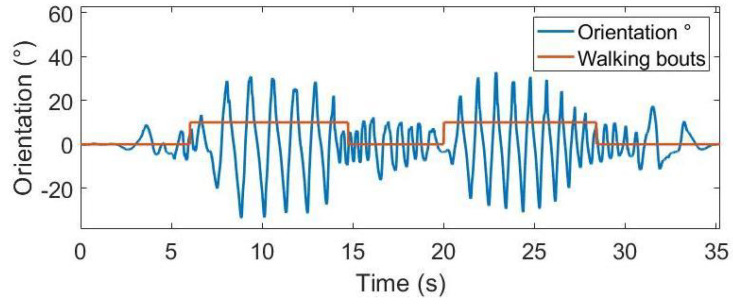
Orientation signal (blue) and walking bouts detected by the algorithm (orange).

The scalogram obtained from the CWT is reported in Figure 3 (restricted in the frequency range 0–1 Hz), where the yellow zones correspond to the walking segments of the signal. In order to identify walking bouts, the intensity profile was computed for each value of the frequency scale; then, the obtained profiles were averaged, and finally, the regions in which the average intensity profile exceeded the standard deviation value were selected. The result of this procedure is reported in Figure 4, where the walking bouts are identified in the orientation signal.

#### 2.3.3. Segmentation and Feature Extraction

In each walking segment of the orientation signal, initial contacts were identified (*ICs*) as the positive orientation signal peaks [36]. Aiming to avoid possible double-peak detection, the orientation signal was low-pass filtered using a second-order zero-lag Butterworth filter, with a cut-off frequency of 2 Hz. In addition, only peaks higher than the signal standard deviation and at least 0.5 s apart were selected. As suggested in [36], final contacts (*FCs*) correspond to the negative peaks following the *ICs*. The acceleration, angular velocity, and orientation recordings were segmented into windows corresponding to strides (i.e., from an *IC* to the subsequent *IC*), in order to prepare the data for the subsequent feature extraction step.

From each stride, a total number of 102 features were extracted from the acceleration, angular velocity, and orientation signal. Features include spatiotemporal gait parameters, and both time- and frequency-domain features. For each stride *i*, stride time, stance time, and swing time were computed as follows:

$$\begin{array}{cccccc} T_{stride} & =IC_{i+1}-IC_{i} & T_{stance} & =FC_{i}-IC_{i} & T_{swing} & =IC_{i+1}-FC_{i} \end{array}$$

Table 4 and Table 5 report the list of features extracted from the time and frequency domain, respectively. The listed features describe different aspects of the gait movement. For instance, Range,Std, and RMS are related to the movement amplitude and intensity; $E_{tot}$ and binEnergy measure the energy content of the signal; Entropy and sEntropy describe movement complexity; DHwidth and DHratio are related to the stride regularity. As far as the spectral features are concerned, they were computed from the Fast Fourier Transform (FFT) of the signal. In order to have homogeneous spectral representations of all strides from all patients, the number of points in which to represent the FFT was set to be $n\;=\;{\bar{T}}_{stride}\cdot F_{s}$, where ${\bar{T}}_{stride}$ is the average stride time found in PDPs [35] and $F_{s}$ is the sample frequency. For strides lasting more than ${\bar{T}}_{stride}$, a small loss of spectral resolution occurs, while for strides lasting less than ${\bar{T}}_{stride}$, some points are added to the FFT, obtained as linear interpolation of the actual data-points. In any case, a spectral resolution of at least 1 Hz is expected, which is adequate for the computation of features listed in Table 5.

### 2.4. PIGD Prediction

This section describes the statistical processing following the extraction of the entire feature set for each patient’s stride, intended to investigate the clinical significance of the extracted features. First, correlation analysis was performed between engineered features extracted from walking bouts and the clinical scores; this was done computing the Pearson correlation coefficient and the corresponding *p*-value for each feature–clinical score pair. Then, a regression model was implemented to predict the PIGD score of PDPs (Section 2.4.1). The analysis was performed in patients both OFF and ON state of therapy, to evaluate the effect of the pharmacological treatment on the performance of the prediction model (Section 2.4.2). Finally, to also evaluate the effect of FOG on model performance, patients were divided based on the clinical presence of FOG (Section 2.4.3).

#### 2.4.1. PIGD Score Regression

The Pearson correlation coefficient (*r*) between the extracted features and the PIGD score was computed in patients both OFF and ON therapy. In order to reduce the dimensionality of the entire feature set (i.e., 102 features), the least significant features (i.e., those with r<0.4) were discarded. To further reduce the set dimensionality, the features were ranked according to their prediction capability. This was done exploiting two different approaches, and evaluating their effect on the final prediction capability.

**Figure 5:**
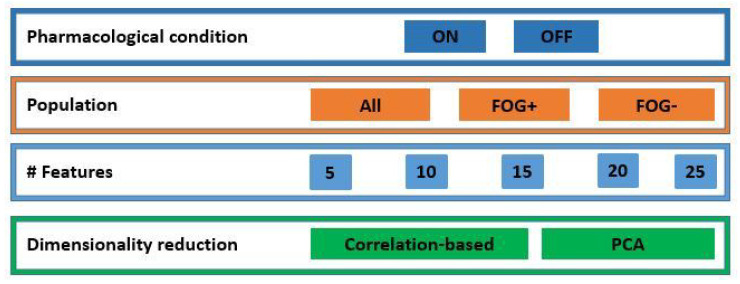
Schematic of the analysis performed. The processing was performed for both pharmacological conditions, different populations, different sizes of the feature set, and different dimensionality reduction methods. ON: under dopaminergic therapy; OFF: not under dopaminergic therapy; FOG: patients with Parkinson’s disease and freezing of gait; FOG−: patients with Parkinson’s disease without freezing of gait; PCA: principal component analysis.

The first approach consisted in sorting the features in descending order of *r*, then selecting the first N features. The second approach made use of principal component analysis (PCA) to reduce the dimensionality of the feature set, keeping only the first N principal components. The parameter N was tuned in the range of 5–25 estimating the effect of the different feature set dimensionality on the model performance. Figure 5 reports a schematic of the entire process.

Feature scaling was applied to each feature using the z-score normalization, which consists in removing the mean value and dividing by the standard deviation. This was done to uniform the feature range, while reducing the effect of possible outliers. Then, range normalization was performed both on the feature set and on the target vector (i.e., PIGD score) to rescale data in the range [0, 1]. Concerning the regression model, a support vector regression (SVR) model [37,38] was implemented. In order to provide a robust performance evaluation, the model was tested employing the leave-one-subject-out (LOSO) cross-validation, which resembles the realistic working condition of the model. It consists in training the model with data from all patients except one, which is used as test. In order to optimize the model parameters, a LOSO-based training-validation procedure was carried out, selecting those parameters providing the best performance on the validation set. Kernel function, kernel scale, and misclassification cost (box-constraint) parameters were optimized for each SVR model, while the margin of tolerance (epsilon parameter) was set to the default value, corresponding to a tenth of the PIGD score standard deviation.

The entire process is described in Algorithm 1. The goodness-of-fit was assessed using the metrics reported in Equation (2).

(2) $$r\;=\;\sqrt{1-\frac{\sum_{i\;=\;1}^{N}{\left(y_{i}-\hat{y}\right)}^{2}}{\sum_{i\;=\;1}^{N}\left(y_{i}-\bar{y}\right)}}\;RMSE\;=\;\sqrt{\frac{1}{N}\sum_{i\;=\;1}^{N}{\left({\hat{y}}_{i}-y_{i}\right)}^{2}}\;MAE\;=\;\frac{1}{N}\sum_{i\;=\;1}^{N}\left|y_{i}-\bar{y}\right|$$

**Algorithm 1** Algorithm for model optimization, validation and test performance evaluation
 **procedure**
optimizedModel(Data,actualScore), performance(Data,actualScore)
▹
  **for**
i←1 to *N*
**do**▹ Perform *N* times test procedure   trainingSet←data from all subjects except for *i*th▹   testSet←data from *i*th subject▹   **for**
kernelfunction←[linear,quadratic,cubic,gaussian]
**do**▹ tune kernel function    **for**
kernelScale←[0.001−1000]
**do**▹ tune kernel scale     **for**
boxConstraint←[0.001−1000]
**do**▹ tune cost parameter      **for**
j←1 to N−1
**do**▹ Perform N−1 times validation procedure       trainingSet←data from trainingSet except for *j*th subject▹       [validationSet]← data from *j*th subject▹       Model←train(model(trainingSet))▹ train model       valPrediction(j)←predict(model(validationSet))▹ predict      **end for**      $RMSE\leftarrow \sqrt{\frac{1}{N-1}\sum_{j=1}^{N-1}{\left(valPrediction-actualScore\right)}^{2}}$▹     **end for**    **end for**   **end for**   [kernelFunction,kernelScale,boxConstraint]←min(RMSE)▹   optimizedModel← model(kernelFunction,kernelScale,boxConstraint)   testPrediction(i)← predict(optimizedModel(testSet))▹ prediction on test set  **end for**  performance← [r,RMSE,MAE](testprediction,actualScore)]▹ test performance  **return**
optimizedModel,performance▹ **end procedure**

The correlation coefficient (r) measures how well the model fits the dependent variable, i.e., how much variability in the dependent variable can be explained by the model; it ranges between 0 and 1, with larger values indicating better performance. Root mean square error (RMSE) and mean absolute error (MAE) are absolute measures of the goodness of fit, providing the entity of deviation from the target values. While MAE treats all errors the same, RMSE gives larger penalization to big prediction errors.

#### 2.4.2. The Effect of L-DOPA

Inertial data from PDPs were divided based on the pharmacological condition. Two independent datasets were obtained from patients OFF and ON state of therapy. The motor condition of patients while OFF and ON was compared performing the Wilcoxon test on the MDS-UPDRS part III and on the PIGD score in the two pharmacological conditions. Then, the analysis reported in Figure 5 was performed, optimizing the model according to Algorithm 1. The performance obtained on patients OFF and ON was compared using different feature set sizes, different dimensionality reduction methods, and optimizing the regression model parameters. Finally, the performance of the model in patients OFF and ON therapy were compared.

#### 2.4.3. The Effect of Freezing of Gait

The dataset was split according to clinical presence of FOG. Then, the Mann–Whitney U-test was used to compare both the clinical scores and the engineered features of FOG+ and FOG− patients. Then, the analysis reported in Figure 5 was performed, optimizing the model according to Algorithm 1. The performance obtained on patients with and without FOG was compared using different feature set sizes, different dimensionality reduction methods, and optimizing the regression model parameters. The entire procedure was carried out for each pharmacological condition. Finally, the the effect of FOG on the model performance was evaluated.

All the experiments were executed in Matlab R2020a, using a personal computer with Microsoft Windows 10, a 2.4 GHz Intel^®^ Core Processor i5-6200, 8 GB RAM and 4 GB GPU.

## 3. Results

### 3.1. Clinical-Behavioural Correlations

**Figure 6:**
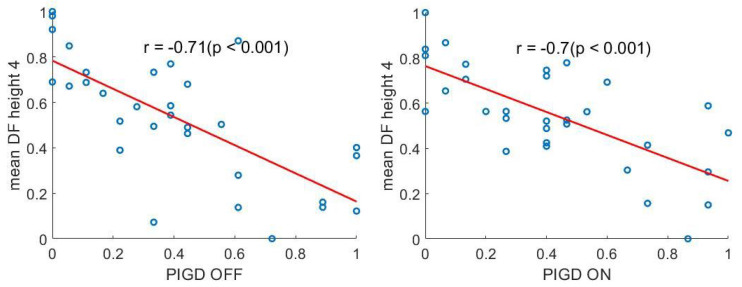
Correlation plot between the average principal harmonic height for the *x*-axis angular velocity (DH height 4) and PIGD score. Data and PIGD score refer to patients OFF (**left**) and ON (**right**) therapy. PIGD: postural instability and gait difficulty.

Pearson correlation analysis showed that most time- and frequency-domain features significantly correlated with PIGD scores. In more detail, as axial motor control worsened, the minimum value of inertial signals increased, whereas maximum and root mean square values of inertial signals, average height of peaks in the time-domain and height of the dominant harmonic decreased. Table 6 summarizes the Pearson correlation coefficients and the respective p-values for different feature-PIGD pairs. Only the most informative features for either therapeutic conditions, i.e., those with a Pearson correlation coefficient with PIGD score larger than 0.5, were included in the table. Figure 6 reports the scatter plots for the average height of the dominant harmonic (mean DH height) versus PIGD score OFF and ON.

### 3.2. PIGD Score Regression

This section reports results from the optimized support vector regression models in LOSO validation. Specifically, Section 3.3 summarizes findings concerning the effect of L-Dopa by comparing regression models in patients OFF and ON therapy. The best model configuration was identified for each pharmacological condition and the performance of the regression models were compared. Section 3.4 reports findings concerning the effect of FOG occurrence, by comparing regression models in *FOG+* and *FOG−*. The best model configuration was extracted for each subgroup of patients and the performance of the regression models were compared.

### 3.3. The Effect of L-DOPA

The Wilcoxon test demonstrated that both UPDRS-part III and the PIGD score were different in patients OFF and ON therapy (*p* < 0.001). Table 7 summarizes the performance of the regression model in terms of correlation coefficient, RMSE, and MAE, in PDPs OFF and ON state of therapy. Results are reported for different sizes of the feature set and different dimensionality reduction methods.

**Figure 7:**
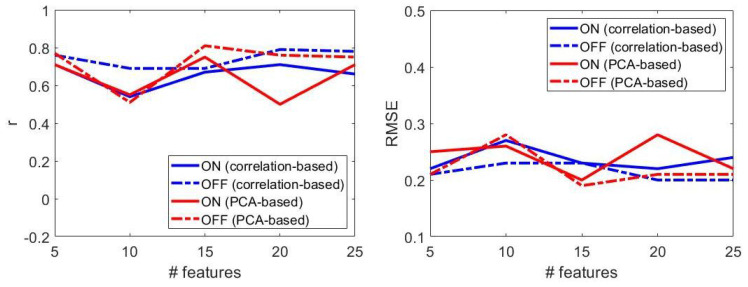
The effect of therapy on the regression model performances, in terms of correlation coefficient (**left**) and root mean square error (**right**). ON: under dopaminergic therapy; OFF: not under dopaminergic therapy.

Based on the results from Table 7, visually reported in Figure 7, the following considerations were derived.

- Model: SVR with linear kernel is selected in 85% of cases; top performances were obtained with linear kernel and small values of box-constraint parameter (i.e., <0.009).
- Number of features: increasing the feature set size did not ensure progressively better performances (Figure 7). Best results were obtained with *n* = 15 features, both for patients OFF and ON therapy.
- Dimensionality reduction: for larger feature set size (i.e., # features > 15), PCA-based dimensionality reduction always implied better results, compared to those attained with correlation-based feature selection (Figure 7). PCA-based dimensionality reduction method led to the best results both for patients OFF and ON therapy.
- Performance: regression models provided better performances in patients OFF than those ON therapy.

**Figure 8:**
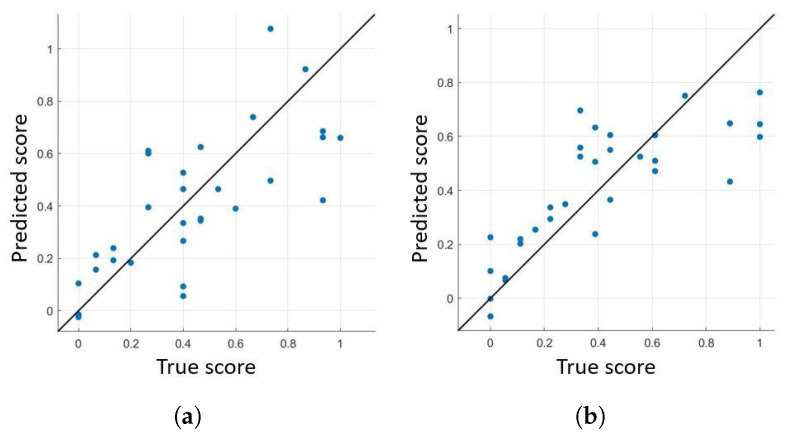
Regression results for patients under different pharmacological condition. Data are plotted using a scatter plot and the regression line is reported as the best fit line. (**a**) Patients under dopaminergic therapy. (**b**) Patients not under dopaminergic therapy.

Consequently, the best regression model parameters were identified for each pharmacological condition. Then, such models were trained on patients ON (OFF) therapy and tested on patients OFF (ON) therapy. This procedure resulted in r = 0.70 (0.67), RMSE = 0.57 (0.42), and MAE = 0.47 (0.15). When the model was tested using LOSO on all available data, regardless of the pharmacological condition, r = 0.64, RMSE = 0.22, and MAE = 0.17 were obtained from an SVR with linear kernel and box-constraint = 0.07. Figure 8 reports the true versus predicted score scatter plot, together with the the best fit line.

### 3.4. The Effect of Freezing of Gait

Table 8 reports the demographic and clinical features of PDPs with and without FOG, along with the significance level computed using the Mann–Whitney U test. In PDPs with FOG, FOG duration was 4.8 ± 2.7 and the total FOG-Q score was 15.4 ± 4.4. As far as concerns the engineered features, the Mann–Whitney U test showed that PDPs with FOG had higher Min and lower Mean, RMS, DHheight, and $E_{tot}$ compared to those without FOG (*p* < 0.001).

**Figure 9:**
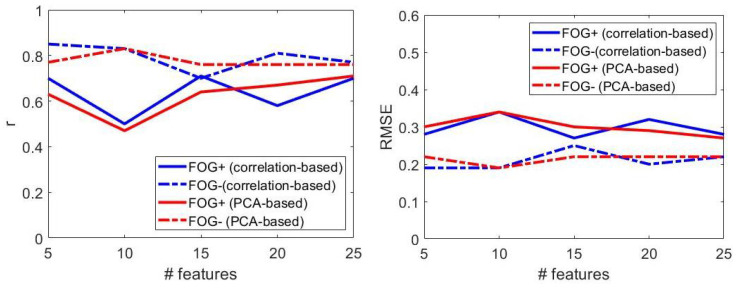
The effect of freezing of gait on the regression model performance, in terms of correlation coefficient (**left**) and root mean square error (**right**). Performance refers to patients under dopaminergic therapy. FOG+: patients with freezing of gait; FOG−: patients without freezing of gait.

Table 9 reports the performance of the optimized regression models for *FOG+* and *FOG−* patients ON state of therapy, visually reported in Figure 9.

From Figure 9, it can be inferred that, when the size of the feature set increased, regression models provided comparable performance in *FOG+* and *FOG−*. This was particularly evident for *n* = 25 features, for which r and RMSE were very similar in the two populations, regardless of the dimensionality reduction method. From the results above, the following considerations were derived.

- Model: SVR with linear kernel is selected in 95% of cases; top performances were obtained with linear kernel and small values of box-constraint parameter (i.e., <0.51).
- Number of features: increasing the feature set size did not ensure progressively better performances (Figure 9). Best results were obtained with *n* = 15 (*n* = 5) features in patients with (without) FOG.
- Dimensionality reduction: correlation-based and PCA-based dimensionality reduction methods provided similar results, regardless of the feature set size (Figure 7).
- Correlation-based dimensionality reduction method led to the best results both for *FOG+* and *FOG−* patients.
- Performance: regression models provided better performances in *FOG−* patients, as evident from larger values of r and lower values of the RMSE (Figure 9).

Based on the considerations above, the dimension of the feature set was set to 15 (5) for *FOG+* (*FOG−*) patients, and the dimensionality reduction method to correlation-based for both populations. Then, the best regression model was trained on *FOG+* (*FOG−*) and tested on *FOG−* (*FOG+*). This procedure resulted in r = 0.34 (0.40), RMSE = 0.43 (0.39), and MAE = 0.37 (0.35), respectively.

The performance gap between PDPs with and without FOG may be due to the different discrimination power of some features in the two populations. From Table 9, it turns out that, for each feature set size, the correlation between top-ranked features and PIGD score is larger in patients without FOG. Top-ranked features for those patients were found to be Min (r = 0.79, *p* = 0.001), vPeaks (r = −0.75, *p* = 0.004), RMS (r = −0.72, *p* = 0.006), hPeaks (r = −0.70, *p* = 0.008) from the *x*-axis orientation signal, and $E_{tot}$ (r = −0.79, *p* = 0.001) from the *x*-axis angular velocity signal. As far as concerns PDPs with FOG, top-ranked features included Min (r = 0.66, *p* = 0.004), DH height (r = −0.62, *p* = 0.008), RMS (r = −0.58, *p* = 0.015), hPeaks (r = −0.58, *p* = 0.016) from the y-axis acceleration signal, and DH height (r = −0.65, *p* = 0.005) from the *x*-axis orientation signal.

**Figure 10:**
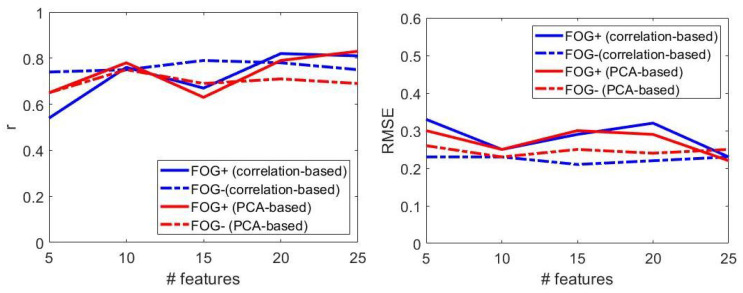
The effect of freezing of gait on the regression model performance, in terms of correlation coefficient (**left**) and root mean square error (**right**). Performance refers to patients not under dopaminergic therapy. FOG+: patients with freezing of gait; FOG−: patients without freezing of gait.

Table 10 reports the performance of the optimized regression models for *FOG+* and *FOG−* OFF state of therapy, visually reported in Figure 10.

Based on the results above, the following considerations were derived.

- Model: SVR with linear kernel is selected in 95% of cases; top performances were obtained with linear kernel both in patients with and without freezing of gait.
- Number of features: increasing the feature set size did not ensure progressively better performances (Figure 10). Best results were obtained with *n* = 25 (*n* = 15) features in patients with (without) freezing of gait.
- Dimensionality reduction: PCA-based (correlation-based) dimensionality reduction was selected for patients with (without) FOG.
- Performance: regression models provided slightly better performances in patients without FOG, in terms of RMSE (Figure 10), independently of the model configuration; performance in terms of *r* depends on the regression model parameters, with best results superior in patients with FOG (Table 10).

Based on the considerations above, the dimension of the feature set was set to 25 (15) and the dimensionality reduction method to the PCA-based (correlation-based) for *FOG+* (*FOG−*). Then, the best regression model were trained on *FOG+* (*FOG−*) and tested on *FOG−* (*FOG+*). This procedure resulted in r = 0.73 (0.69), RMSE = 0.36 (0.33), and MAE = 0.25 (0.25), respectively.

Table 11 reports all the results obtained for each population under investigation and for each pharmacological condition; results were obtained using LOSO test. As evident from the table, model performance improved when considering separately patients in different pharmacological conditions. Concerning the effect of FOG, if the model is specifically trained on *FOG+* and *FOG−* separately, the performance significantly improves in patients without FOG while ON state of therapy and in patients with FOG while OFF therapy. Table 12 reports the results obtained by training and testing the regression model on different populations (i.e., ON versus OFF therapy, FOG+ versus FOG−). Prediction errors provided by the global model (i.e., the regression model trained and validated on all subjects, independently of the pharmacological condition and of freezing of gait) were compared to those obtained using different models for each pharmacological condition separately. The Wilcoxon test proved that the difference in prediction errors was not statistically significant (*p* = 0.074); thus, a single model may be used to estimate the PIGD score. On the other hand, training the model on specific subgroups (e.g., patients with FOG, patients ON therapy) and testing on different subgroups led to a large performance impairment, as evident from RMSE values reported in Table 12. Summarizing these findings, it is possible to implement a very general algorithm, but attention should be paid to collect a very general dataset, including patients in different pharmacological conditions, as well as patients with and without FOG.

**Figure 11:**
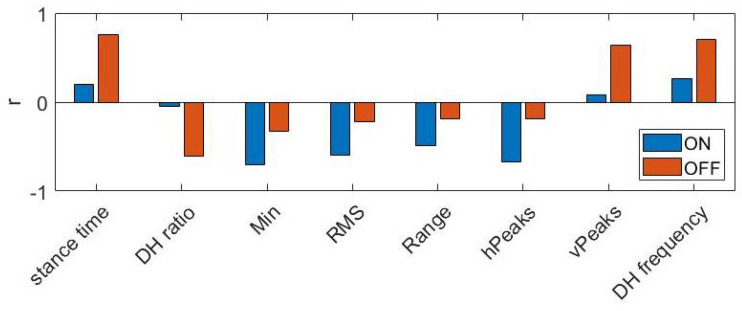
Pearson correlation coefficient between features and PIGD score. DH ratio and DH frequency refer to the component $\theta_{x}$; Min and vPeaks refer to the component $\omega_{x}$; RMSE and Range refer to the component $\alpha_{y}$.

The large prediction errors observed when training and testing the model on different populations may be due to the different discrimination power of some features. As can be observed in Figure 11, the sensibility of some features to changes in PIGD score depends on the pharmacological condition. Some features were found to exhibit strong correlation with PIGD in patients ON therapy but not in patients OFF therapy, and vice versa. The same behaviour can be observed when training and testing the regression model on patients with and without FOG, while ON therapy. As previously reported when discussing Table 9, top-ranked features were different in FOG+ and FOG− patients, thus, the prediction model performance worsens when trained and tested on different populations.

Finally, from Table 12, it can be noticed that performance did not get significantly impaired when training the model with data from FOG+ (FOG−) patients and testing on data from FOG− (FOG+) patients, while OFF therapy. In this case, common top-ranked features included minimum value, root mean square value, and average height of peaks from the orientation signal; average value of the angular velocity signal; height of the dominant harmonic from the acceleration signal along the *y*-axis.

## 4. Discussion

Machine-learning algorithms can reliably predict PIGD scores in PDPs during gait through sensor-based recordings. In this study, a homogeneous cohort of PDPs was recruited and the PIGD scores were calculated in patients OFF and ON therapy, according to standardized clinical procedures. To further control for clinical biases, patients were allocated to the FOG or non-FOG group according to the direct observation of FOG episodes rather than only considering patients’ records. Furthermore, to maximize the prediction performance, many time- and frequency-domain features were computed in addition to classical spatio-temporal parameters routinely used in gait analysis studies. Lastly, a comprehensive statistical analysis was provided on both clinical scores and engineered features, to provide deeper insights into the capability of features to measure axial motor impairment in PDPs.

Significant correlations were found between specific sensor-based variables in the time-as well as frequency-domain and PIGD scores, suggesting that higher PIGD scores are associated with greater kinematic abnormalities during gait in PDPs. In more detail, higher axial motor impairment measured with PIGD was associated with greater abnormalities in movement amplitude, intensity, and regularity in PDPs. In line with these findings, the authors of [23] found significant associations of PIGD scores with sensor-based measures, including the number of walking bouts, gait speed and sway area. These findings also agree with previous studies showing higher impairment of spatio-temporal gait parameters in PDPs presenting a PIGD phenotype with more severe axial dysfunction than those with a tremor-dominant phenotype [39,40]. Accordingly, our clinical-behavioural correlations lay the foundations for elaborating PIGD prediction models based on the considered time- and frequency-domain features.

When considering PIGD prediction with respect to L-Dopa intake, regression models had better performances in PDPs OFF than those ON state of therapy (*p* = 0.002). The finding of better performance in PDPs OFF with respect to those ON therapy is in line with previous results, reporting variable accuracy of ML algorithms in the sensor-based assessment of motor disorders in PDPs in different pharmacological conditions [41]. Indeed, L-Dopa significantly changes spatiotemporal stride parameters and, accordingly, acts on the ML performance when measuring gait in PD [41,42]. Since the PIGD score consists of several items reflecting both postural and walking abilities, a possible explanation for different ML performances in patients OFF and ON therapy relies on the heterogeneous L-Dopa sensitivity of balance and gait in PD. Indeed, unlike gait, L-Dopa usually does not substantially impact balance in PD [7,43,44]. Therefore, we hypothesize that PIGD scores are more accurately predicted in patients OFF than those ON state of therapy owing to a more similar trend of postural and walking abilities in patients not under dopaminergic therapy. The performance of our regression models in PDPs both OFF and ON therapy were higher than those reported in a previous study testing PDPs only ON therapy [23]. Moreover, while in [23] different activities (i.e., gait, turn, stance) were analysed to provide the final output, in the present study only features extracted from walking bouts were used to predict the PIDG score. Our results suggested that it is possible to implement a single regression model capable of predicting PIGD in PDPs, regardless of the therapeutic condition and the presence of freezing of gait. However, data should be collected from a heterogeneous cohort of PDPs, under different pharmacological conditions. When models were trained on a subgroup of patients (i.e., patients with FOG, patients ON therapy), impaired performance was observed when testing on a different subgroup.

Several time- and frequency-domain features, such as Min, RMS, Mean and DHheight, were found to have different sensibility in patients with and without FOG, a finding fully in agreement with previous studies demonstrating worse continuous gait abnormalities in PD patients with FOG than those without FOG, also outside FOG episodes [45,46,47]. Moreover, different time- and frequency-domain features also explain another relevant finding of this study, that is, models trained in patients OFF therapy do not perform well in patients ON therapy and vice versa. It is likely that, in addition to changes of continuous gait parameters and pharmacological condition, the unpredictable and sudden appearance of FOG affects the walking pattern in PDPs, worsening ML performances. In addition, despite the direct impact on gait, FOG is not included in the calculation of the PIGD score and, accordingly, is not considered for the assessment of axial impairment when using this standardized clinical index.

Unlike the only previous study predicting PIGD scores in PD using three sensing devices [23], only a single, small, and lightweight wearable inertial sensor placed on the thigh was here used, providing a little demanding and unobtrusive solution for everyday application in free-living settings. However, future studies are necessary to clarify the technical feasibility of applying the proposed ML algorithms to data recorded through smartphones in non-supervised environments.

In this study, the experimental tools, including wearable sensors and ML algorithms, were already largely used and validated [48,49,50,51]. The novelty of our study primarily relies on the application of these tools to overcome the clinical need for quantitative measures reflecting axial impairment in PD. However, when considering the findings of this study, the lack of validation on an independent test set is a possible limitation to be accounted for. Indeed, data from thirty-one subjects were used for the analysis. Accordingly, additional studies are necessary to reproduce these findings in larger cohorts of patients.

## 5. Conclusions

A single inertial sensor placed on the thigh may be a feasible wearable solution for the remote assessment of axial impairment in PD by predicting the PIGD score through dedicated ML algorithms. When implementing prediction models of PIGD scores, patients’ pharmacological conditions and FOG occurrence are significant clinical variables to be considered. The use of an unobtrusive sensing system composed of a single inertial sensor supports the future adoption of commonly available smartphones, embedding inertial systems, for the long-term motor assessment in PD. Accordingly, future studies will address the need for collecting additional sensor-based data in PDPs to further implement subject-dependent prediction models. In addition, data collection will be performed directly in unsupervised conditions to monitor free-living daily activities and get more ecological measures.

## Figures and Tables

**Table 1:** Demographic and clinical features of patients enrolled in the present study (mean ± standard deviation). H&Y: Hoehn and Yahr.

# Patients (Male)	Age (Years)	Disease Duration (Years)	H&Y
31 (23)	71.9 ± 6.9	10.9 ± 5.9	2.4 ± 0.8

**Table 2:** Standardised scales and scores of patients enrolled in the present study (mean ± standard deviation). MMSE: mini-mental state examination; FAB: frontal assessment battery; HAM-D: Hamilton depression rating scale; BAI: Beck anxiety inventory; LEDD: L-Dopa equivalent daily dose; MDS-UPDRS-III: Movement Disorder Society—unified Parkinson’s disease rating scale part III; OFF: not under dopaminergic therapy; ON: under dopaminergic therapy; PIGD: postural instability/gait difficulty score.

MMSE	FAB	HAM-D	BAI	LEDD (mg)	MDS-UPDRS-III OFF (ON)	PIGD OFF (ON)
28.1 ± 1.9	14.7 ± 2.8	12.9 ± 6.8	11.8 ± 7.5	819 ± 406	35.9 ± 13.9 (27.9 ± 13.7)	7.3 ± 5.7 (6.3 ± 4.6)

**Table 3:** Inertial sensors technical characteristics.

Sensor	Range	Sensitivity	Sample Rate
Accelerometer	±2 g	61 μg/LSB	60 Hz
Gyroscope	±245 dps	8.75 mdps/digit	60 Hz

**Table 4:** List of time-domain features extracted in the present study, together with equations and some brief explanations. α: acceleration; ω: angular velocity; θ: orientation.

ID	Feature	Component	Number	Equation	Explanation
1	Min	$\alpha_{y},\alpha_{z},\omega_{x},\theta_{x}$	4	-	minimum value
2	Max	$\alpha_{y},\alpha_{z},\omega_{x},\theta_{x}$	4	-	maximum value
3	Mean	$\alpha_{y},\alpha_{z},\omega_{x},\theta_{x}$	4	$\bar{x}\;=\;\frac{1}{N}\sum_{i=1}^{N}x_{i}$	average value
4	*Std*	$\alpha_{y},\alpha_{z},\omega_{x},\theta_{x}$	4	$\sigma_{x}\;=\;\sqrt{\frac{1}{N}\sum_{i=1}^{N}{\left(x_{i}-\bar{x}\right)}^{2}}$	standard deviation
5	*RMS*	$\alpha_{y},\alpha_{z},\omega_{x},\theta_{x}$	4	$x_{RMS}\;=\;\sqrt{\frac{1}{N}\sum_{i=1}^{N}x_{i}^{2}}$	root mean square value
6	*Range*	$\alpha_{y},\alpha_{z},\omega_{x},\theta_{x}$	4	$r_{x}$ = $x_{max}-x_{min}$	range of values
7	*Entropy*	$\alpha_{y},\alpha_{z},\omega_{x},\theta_{x}$	4	$E_{x}\;=\;x\log\left(x+\epsilon \right),\epsilon \;=\;10^{-5}$	Shannon signal entropy
8	nPeaks	$\alpha_{y},\alpha_{z},\omega_{x},\theta_{x}$	4	-	number of peaks higher than Std
9	hPeaks	$\alpha_{y},\alpha_{z},\omega_{x},\theta_{x}$	4	-	average height of nPeaks
10	vPeaks	$\alpha_{y},\alpha_{z},\omega_{x},\theta_{x}$	4	-	standard deviation of hPeaks
11	Zc	$\alpha_{y},\alpha_{z},\omega_{x},\theta_{x}$	4	-	zero-crossing rate
12	Corr	$\alpha_{y},\alpha_{z},\omega_{x},\theta_{x}$	7	$r\left(i,j\right)\;=\;\frac{cov(i,j)}{\sigma (i)\sigma (j)}$	correlation between axis pair

**Table 5:** List of spectral-domain features extracted in the present study, together with equations and some brief explanations. α: acceleration; ω: angular velocity; θ: orientation.

ID	Feature	Component	Number	Equation	Explanation
13	DH frequency	$\alpha_{y},\alpha_{z},\omega_{x},\theta_{x}$	4	-	frequency of the principal harmonic
14	DH height	$\alpha_{y},\alpha_{z},\omega_{x},\theta_{x}$	4	-	amplitude of the principal harmonic
15	DH width	$\alpha_{y},\alpha_{z},\omega_{x},\theta_{x}$	4	-	width of the principal harmonic
16	$E_{tot}$	$\alpha_{y},\alpha_{z},\omega_{x},\theta_{x}$	4	$\sum_{f\;=\;1}^{Fs/2}X_{f}$	total signal energy
17	DH ratio	$\alpha_{y},\alpha_{z},\omega_{x},\theta_{x}$	4	-	ratio between the energy of the principal harmonic and $E_{tot}$
18	*sEntropy*	$\alpha_{y},\alpha_{z},\omega_{x},\theta_{x}$	4	$X_{f}\log\left(X_{f}+\epsilon \right)$	Shannon entropy of the signal FFT
19	*binEnergy*	$\alpha_{y},\alpha_{z},\omega_{x},\theta_{x}$	24	-	ratio between energy in specific frequency bands and $E_{tot}$

**Table 6:** Correlation between engineered features and PIGD score. Results are reported in terms of Pearson correlation coefficient and relative *p*-value. For each feature, significant components are reported. α: acceleration; ω: angular velocity; θ: orientation.

Feature	Component	Pearson Correlation Coefficient (*p*-Value)	
		PIGD OFF	PIGD ON
Min	$\alpha_{y}$	0.54 (0.002)	0.58 (<0.001)
Mean	$\omega_{x}$	0.64 (<0.001)	0.54 (0.002)
RMS	$\alpha_{y},\omega_{x}$	−0.67 (<0.001), −0.74 (<0.001)	−0.57 (0.002), −0.72 (<0.001)
hPeaks	$\alpha_{y},\omega_{x}$	−0.70 (<0.001), −0.74 (<0.001)	−0.58 (<0.001), −0.60 (<0.001)
DH height	$\alpha_{y}\omega_{x},\theta_{x}$	−0.59 (<0.001), −0.71 (<0.001), −0.69 (<0.001)	−0.57 (<0.001), −0.70 (<0.001), −0.65 (<0.001)

**Table 7:** Performance of regression models in different pharmacological conditions using leave-one-subject-out validation. Results were obtained for different sizes of the feature set and different dimensionality reduction methods. Best performances are marked with bold type. ON: under dopaminergic therapy; OFF: not under dopaminergic therapy; var: total variance explained; r: correlation coefficient; SVR: support vector regression; RMSE: root mean square error; MAE: mean absolute error.

# Features	Therapy	Dimensionality Reduction		SVR Parameters			Performance		
		Method	Value	Kernel	Kernel Scale	Box Constraint	r	RMSE	MAE
5	ON	r (min–max)	0.65–0.72	linear	-	10.9	0.71	0.22	0.18
		var (%)	82.9	linear	-	0.09	0.71	0.25	0.20
	OFF	r (min–max)	0.76–0.77	gaussian	1.41	2.67	0.76	0.21	0.18
		var (%)	77.3	linear	-	0.006	0.77	0.21	0.16
10	ON	r (min–max)	0.58–0.72	linear	-	378.6	0.54	0.27	0.22
		var (%)	93.0	linear	-	0.07	0.55	0.26	0.20
	OFF	r (min–max)	0.74–0.77	linear	-	0.35	0.69	0.23	0.19
		var (%)	88.7	linear	-	1.91	0.51	0.28	0.23
15	ON	r (min–max)	0.56–0.72	linear	-	0.003	0.67	0.23	0.19
		var (%)	97.5	linear	-	0.009	**0.75**	**0.20**	**0.16**
	OFF	r (min–max)	0.68–0.77	gaussian	449.69	253.51	0.69	0.23	0.19
		var (%)	94.7	linear	-	0.001	**0.79**	**0.19**	**0.15**
20	ON	r (min–max)	0.55–0.72	linear	-	0.002	0.71	0.22	0.16
		var (%)	99.2	linear	-	641.6	0.5	0.28	0.24
	OFF	r (min–max)	0.66–0.77	linear	-	0.004	0.79	0.20	0.15
		var (%)	97.9	gaussian	40.06	0.87	0.76	0.21	0.15
25	ON	r (min–max)	0.52–0.72	linear	-	0.005	0.66	0.24	0.19
		var (%)	99.8	linear	-	0.003	0.71	0.22	0.16
	OFF	r (min–max)	0.62–0.72	linear	-	0.004	0.78	0.20	0.15
		var (%)	99.5	cubic	-	0.19	0.75	0.21	0.16

**Table 8:** Demographic and clinical features of patients enrolled in the present study (mean ± standard deviation). H&Y: Hoehn and Yahr; FOG: freezing of gait; FOG-Q: freezing of gait questionnaire.

Group	# Patients (Male)	Age (Years)	MDS-UPDRS-III OFF (ON)	PIGD OFF (ON)
FOG+	17 (13)	72.0 ± 7.6	40.9 ± 13.2 (32.9 ± 14.1)	11.2 ± 4.5 (9.6 ± 3.4)
FOG−	14 (10)	71.8 ± 6.4	29.7 ± 12.3 (21.9 ± 10.8)	2.6 ± 2.5 (2.4 ± 2.3)
*p*	0.353 (0.531)	0.811	0.054 (0.030)	<0.001 (<0.001)

**Table 9:** Performance of regression models in patients with (FOG+) and without (FOG−) freezing of gait, both under dopaminergic therapy. Results were obtained for different feature set size and different dimensionality reduction methods. Best performances are marked with bold type. RMSE: root mean square error; r: correlation coefficient; MAE: mean absolute error.

# Features	Group	Dimensionality Reduction		SVM Parameters			Performance		
		Method	Value	Kernel	Kernel Scale	Box Constraint	r	RMSE	MAE
5	FOG+	r (min–max)	0.58–0.69	linear	-	0.007	0.7	0.28	0.22
		var (%)	85.2	linear	-	0.02	0.63	0.30	0.25
	FOG−	r (min–max)	0.77–0.84	linear	-	0.07	**0.85**	**0.19**	**0.13**
		var (%)	82.1	linear	-	0.007	0.77	0.22	0.15
10	FOG+	r (min–max)	0.55–0.69	linear	-	1.69	0.5	0.34	0.26
		var (%)	96.6	linear	-	0.03	0.47	0.34	0.29
	FOG−	r (min–max)	0.69–0.84	linear	-	0.02	0.83	0.19	0.15
		var (%)	96.5	linear	-	0.004	0.83	0.19	0.15
15	FOG+	r (min–max)	0.53–0.69	linear	-	0.51	**0.71**	**0.27**	**0.21**
		var (%)	99.8	linear	-	0.06	0.64	0.30	0.24
	FOG−	r (min–max)	0.66–0.84	quadratic	-	0.019	0.7	0.25	0.20
		var (%)	99.8	linear	-	0.15	0.76	0.22	0.18
20	FOG+	r (min–max)	0.50–0.69	linear	-	0.007	0.58	0.32	0.28
		var (%)	99.9	linear	-	0.46	0.67	0.29	0.23
	FOG−	r (min–max)	0.64–0.84	linear	-	0.004	0.81	0.20	0.25
		var (%)	99.9	linear	-	0.01	0.76	0.22	0.17
25	FOG+	r (min–max)	0.46–0.69	linear	-	0.014	0.7	0.28	0.24
		var (%)	99.9	linear	-	4.02	0.71	0.27	0.22
	FOG−	r (min–max)	0.62–0.84	linear	-	0.02	0.77	0.22	0.19
		var (%)	99.95	linear	-	85.9	0.76	0.22	0.18

**Table 10:** Performance of regression models in patients with (FOG+) and without (FOG−) freezing of gait, both not under dopaminergic therapy. Results were obtained for different feature set sizes and different dimensionality reduction methods. Best performances are marked with bold type. RMSE: root mean square error; r: correlation coefficient; MAE: mean absolute error.

# Features	Group	Dimensionality Reduction		SVM Parameters			Performance		
		Method	Value	Kernel	Kernel Scale	Box Constraint	r	RMSE	MAE
5	FOG+	r (min–max)	0.58–0.65	linear	-	2.7	0.54	0.33	0.29
		var (%)	83.1	gaussian	69.3	2.3	0.65	0.30	0.25
	FOG−	r (min–max)	0.70–0.76	linear	-	0.01	0.74	0.23	0.16
		var (%)	83.5	linear	-	0.06	0.65	0.26	0.20
10	FOG+	r (min–max)	0.55–0.65	linear	-	0.93	0.76	0.25	0.18
		var (%)	95.7	linear	-	0.004	0.78	0.25	0.22
	FOG−	r (min–max)	0.69–0.76	linear	-	83.9	0.75	0.23	0.18
		var (%)	97.2	linear	-	0.003	0.75	0.23	0.15
15	FOG+	r (min–max)	0.52–0.65	linear	-	0.03	0.67	0.29	0.24
		var (%)	99.7	linear	-	118.5	0.63	0.30	0.26
	FOG−	r (min–max)	0.63–0.76	linear	-	0.006	**0.79 **	**0.21**	**0.15**
		var (%)	99.6	linear	-	0.002	0.69	0.25	0.16
20	FOG+	r (min–max)	0.50–0.65	linear	-	0.37	0.82	0.22	0.19
		var (%)	99.8	linear	-	0.05	0.79	0.24	0.21
	FOG−	r (min–max)	0.61–0.76	linear	-	0.009	0.78	0.22	0.14
		var (%)	99.8	linear	-	0.002	0.71	0.24	0.16
25	FOG+	r (min–max)	0.48–0.65	linear	-	621.2	0.81	0.23	0.19
		var (%)	99.9	linear	-	24.2	**0.83**	**0.22**	**0.19**
	FOG−	r (min–max)	0.59–0.76	linear	-	0.69	0.75	0.23	0.16
		var (%)	99.9	linear	-	0.12	0.69	0.25	0.17

**Table 11:** Performance of regression models on different populations under different pharmacological conditions. ON: under dopaminergic therapy; OFF: not under dopaminergic therapy; FOG+: patients with freezing of gait; FOG−: patients without freezing of gait.

Therapy	FOG	Performance		
		r	RMSE	MAE
All	All	0.64	0.22	0.17
ON	All	0.75	0.20	0.16
	FOG+	0.71	0.27	0.21
	FOG−	0.85	0.19	0.13
OFF	All	0.79	0.19	0.15
	FOG+	0.83	0.22	0.19
	FOG−	0.79	0.21	0.15

**Table 12:** Performance of the regression model for different combination of training and test samples. ON: under dopaminergic therapy; OFF: not under dopaminergic therapy; FOG+: patients with freezing of gait; FOG−: patients without freezing of gait.

Training Sample	Testing Sample	Performance		
		r	RMSE	MAE
ON	OFF	0.70	0.57	0.47
OFF	ON	0.67	0.42	0.15
FOG+ (ON)	FOG− (ON)	0.34	0.43	0.37
FOG− (ON)	FOG+ (ON)	0.40	0.39	0.35
FOG+ (OFF)	FOG− (OFF)	0.73	0.36	0.25
FOG− (OFF)	FOG+ (OFF)	0.69	0.33	0.25

## Data Availability

The data presented in this study are available on request from the corresponding author.

## Abbreviations

The following abbreviations are used in this manuscript:

BAI	Beck Anxiety Inventory
CWT	Continuous Wavelet Transform
FAB	Frontal Assessment Battery
FFT	Fast Fourier Transform
FOG	Freezing of Gait
FOG-Q	Freezing of Gait Questionnaire
FOG+	patients with Freezing of Gait
FOG−	patients without Freezing of Gait
HAM-D	Hamilton Depression rating scale
H&Y	Hoehn and Yahr scale
IMU	Inertial Measurement Unit
LEDD	Levodopa Equivalent Daily Dose
LOSO	Leave-One-Subject-Out
LSB	Least Significant Bit
ML	Machine Learning
MAE	Mean Square Error
MDS-UPDRS	Movement Disorder Society—Unified Parkinson’s Disease Rating Scale
MMSE	Mini-Mental State Examination
ON	under dopaminergic therapy
OFF	not under dopaminergic therapy
PIGD	Postural Instability and Gait Difficulty
PD	Parkinson’s Disease
PDPs	Patients with Parkinson’s Disease
RMSE	Root Mean Square Error
TUG	Timed Up and GO
